# Cardiovascular comorbidities in psoriatic arthritis: state of the art

**DOI:** 10.1177/1759720X241274537

**Published:** 2024-09-14

**Authors:** Mrinalini Dey, Elena Nikiphorou

**Affiliations:** Centre for Rheumatic Diseases, King’s College London, Weston Education Centre, London, UK; Centre for Rheumatic Diseases, King’s College London, Weston Education Centre, Cutcombe Road, London SE5 9RJ, UK; Rheumatology Department, King’s College Hospital, London, UK

**Keywords:** cardiovascular, comorbidity, inflammatory arthritis, multimorbidity, psoriatic arthritis

## Abstract

Psoriatic arthritis (PsA) is a complex multi-system immune-mediated condition, characterised by a high comorbidity burden, one of the most prevalent of which is cardiovascular disease (CVD), affecting up to 80% of patients. This narrative review explores the current understanding of cardiovascular comorbidities in PsA, focusing on mechanistic pathways, risk assessment, and the impact of treatment choices on cardiovascular health. Here, we outline the role of inflammatory cytokines, immune system dysregulation, and genetic predispositions in PsA, not only as drivers of musculoskeletal manifestations but also atherosclerosis and endothelial dysfunction, giving rise to cardiovascular pathology. Given these insights, accurately assessing and predicting cardiovascular risk in PsA patients is a critical challenge. This review evaluates traditional risk calculators as well as innovative biomarkers and imaging techniques, emphasising their utility and limitations in capturing the true cardiovascular risk profile of PsA patients. There are multiple complexities surrounding the treatment of PsA in the context of concurrent CVD, and therapeutic choices must carefully balance efficacy in managing PsA symptoms with the potential cardiovascular implications. A multidisciplinary approach, integrating dermatological, rheumatological, and cardiological perspectives, amongst others, to optimise patient outcomes, is key. Overall, a heightened clinical awareness and research focus on cardiovascular comorbidities in PsA is warranted, aiming to refine risk assessment strategies and therapeutic interventions that holistically address the multifaceted needs of patients with PsA.

## Introduction

**Figure 1.**
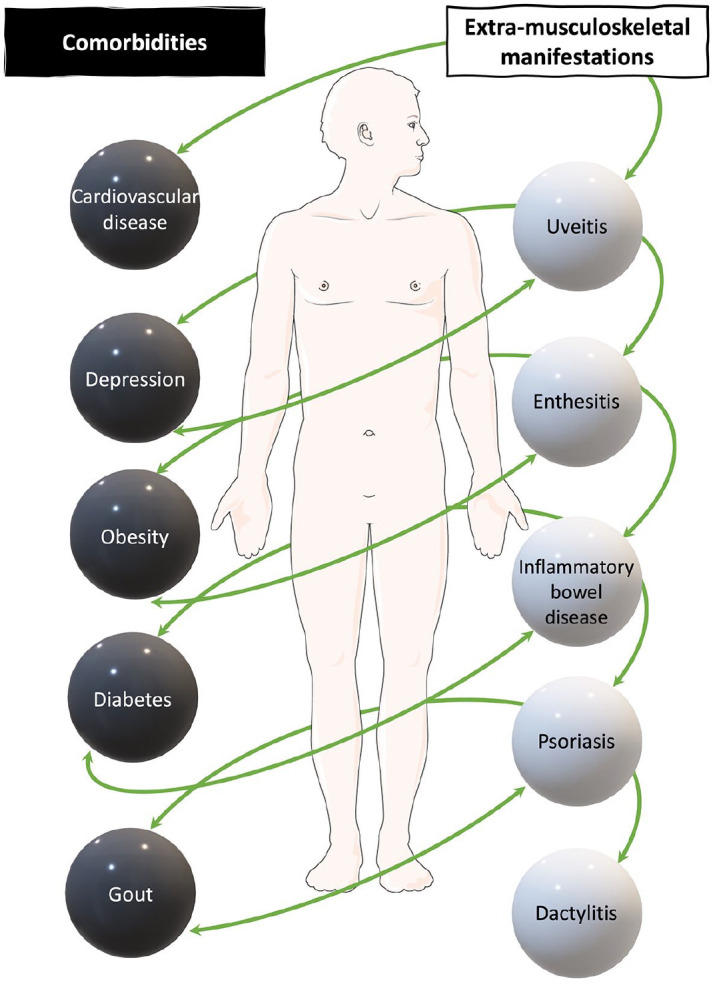
An overview of examples of common comorbidities and extra-musculoskeletal manifestations in patients with psoriatic arthritis. Obesity, type 2 diabetes mellitus and cardiovascular disease, as well as other conditions such as hyperlipidaemia may be present as part of a metabolic syndrome. All, some or none of these comorbidities and/or extra-musculoskeletal manifestations may be present in an individual with psoriatic arthritis.

Psoriatic arthritis (PsA) is a chronic inflammatory immune-mediated condition, affecting approximately 112 in 100,000 adults worldwide.^1^ It is a multi-system disorder, which not only affects approximately 30% of people with psoriasis but is also associated with a high comorbidity burden and extra-musculoskeletal manifestations (EMMs)^2,3^ (Figure 1). Forty percent of patients have three or more comorbidities.^4^ PsA can therefore be highly debilitating, resulting in limitations in function, engagement with work and social interaction, and quality of life.^5,6^ EMMs include uveitis and inflammatory bowel disease, as well as nail, enthesial and skin involvement. However, it is increasingly recognised that people with PsA are at increased risk of developing additional chronic conditions, known as comorbidities, including cardiovascular disease (CVD), metabolic syndrome and mental health disorders.^7–9^

Cardiovascular comorbidities in PsA are associated with increased morbidity and mortality, conferring further complexities in managing the condition, likely due to an interplay of systemic inflammation, metabolic dysregulation and shared genetic predispositions.^10^ In a recent cohort study, 82% of people with early PsA were identified as having cardiovascular risk factors at baseline, 64% had dyslipidaemia and 40% were obese.^11^ In recent years, the European Alliance of Associations of Rheumatology (EULAR) has advocated and published dedicated guidelines for optimised management of CVD in patients with inflammatory arthritis, emphasising its prevalence and importance for clinical decision-making.^12^ The 2022 British Society for Rheumatology guidelines for the management of PsA advises clinicians to consider CVD when making treatment decisions due to the risk of adverse events, as well as addressing modifiable risk factors.^13^

In this review, we aim to delineate the current understanding of cardiovascular comorbidities associated with PsA, explore the epidemiological evidence, discuss implications for clinical outcomes and quality of life, and identify gaps for future research.

## Metabolic syndrome: At the heart of cardiovascular risk in PsA?

Patients with certain inflammatory joint disorders, including PsA, have an increased risk of CVD, as well as metabolic syndrome (including obesity, type 2 diabetes mellitus (T2DM)), compared to the general population.^12^ However, the incidence of cardiovascular risk factors (e.g. dyslipidaemia, obesity) in PsA is significantly elevated, not only compared to the general population but also appears to be elevated compared to people with rheumatoid arthritis.^10,14^ Therefore, there is an urgency to improve our understanding of this observed association and management to reduce this relatively increased morbidity and mortality.

**Figure 2.**
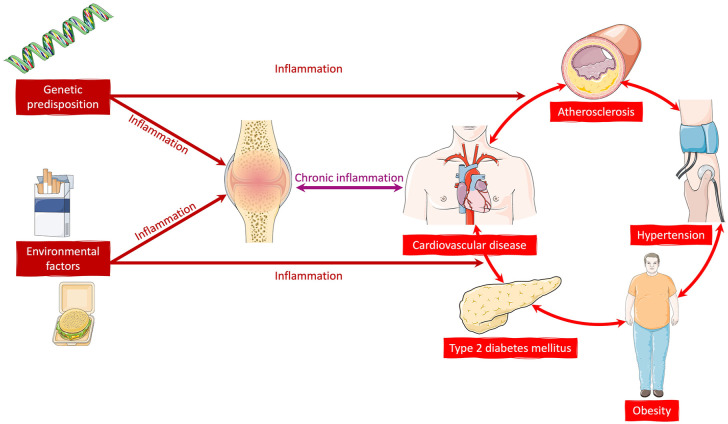
Mechanistic links and inflammatory pathways perpetuating metabolic syndrome and cardiovascular disease in patients with psoriatic arthritis.

The majority of newly diagnosed patients with PsA have a greater than 10% risk of CVD within 10 years of diagnosis, with a recent cohort study identifying 82% of newly diagnosed patients as having cardiovascular risk factors.^11,15^ Over time, this risk accumulates along with an overall increase in comorbidity burden due to not only the presence of PsA and its treatment but also increasing age and presence of other relevant environmental and genetic risk factors, such as smoking and diet (Figure 2). The most prevalent comorbidities in those with long-standing PsA are hypertension, metabolic syndrome, obesity, hyperlipidaemia and CVD, with a pooled prevalence of 19%–34% in a recent meta-analysis.^16^ The presence of these comorbidities is associated with significant morbidity, including poorer quality of life and increased discontinuation of treatment, as well as more severe joint disease.^10,16^

This increased prevalence of metabolic syndrome leads to an increased risk of CVD, including myocardial infarction, not only compared to the general population but also compared to patients with psoriasis alone.^11,17–19^ In one cohort of patients newly diagnosed with PsA, 64% were noted to have dyslipidaemia, while 40% were obese.^11^ Meta-analysis data have shown people with PsA to be at increased risk of having concurrent T2DM (OR 2.18, 95% CI 1.36–3.50).^20^ Even compared to patients with psoriasis alone, there is up to a 50% increased risk of T2DM in people with PsA.^18^ In fact, when comparing the prevalence of hypertension, obesity, hyperlipidaemia, T2DM and cardiovascular events in people with PsA to psoriasis alone, the odds ratio ranges from 1.5 to 2.6.^21^ Geographical and sociocultural aspects also interact with the biological factors in driving the cardiovascular risk. For example, a recent European cohort study found the prevalence of hypertension, T2DM and CVD to be higher in Italian patients with PsA compared to Belgians.^17^

When considering obesity, 30%–40% of people with PsA have this condition which is strongly associated with CVD and wider complications.^11,21^ While obesity may be a risk factor for the development of PsA, it may also be a consequence of the pro-inflammatory state and relative immobility arising from joint disease.^22,23^ Conversely, weight loss not only potentially contributes to a diminished CVD risk but can also improve the response to treatment such as tumour necrosis factor inhibitors (TNFis), thus conferring benefits for both disease activity and CVD outcomes.^24^

## Cardiovascular events in people with PsA

Early studies in outpatient cohorts of patients with PsA, based in the United Kingdom and Canada, identified CVD to be the leading cause of mortality in this population, with 36%–38% of deaths attributable to CVD.^25–27^ Data from the Toronto group found young patients in particular to have an increased mortality risk, associated with the presence of CVD, as well as elevated acute phase reactants and lower education level.^28^ Of a cohort of 611 patients with PsA followed up for 12 months, 8.2% had had at least one cardiovascular event, including myocardial infarction, cardiomyopathy, congestive heart failure and cerebrovascular disease.^21^ A more recent cohort study in Sweden has demonstrated all-cause mortality in patients with PsA to be 10% higher than in the general population, driven by excess comorbidity and more pronounced in women and patients with greater duration of disease.^29^ CVD was also the leading cause of death in this study.

It is noteworthy that an increased prevalence of CVD has been noted in psoriasis, compared to healthy individuals, and the severity of skin disease correlates with the development of subclinical atherosclerosis.^10,30,31^ Furthermore, psoriasis is an independent risk factor for myocardial infarction.^30^ This is likely to be due to a combination of a pro-inflammatory state and environmental risk factors – possible mechanisms are discussed later in this review. Targeted approaches to managing risks, accounting for both biological and non-biological factors, are clearly required.

## Mechanistic pathways in the development of cardiovascular comorbidities in PsA

Inflammation may be an independent risk factor of CVD in people with PsA. Epidemiological studies have demonstrated that the burden of inflammation (measured by C-reactive protein (CRP) and erythrocyte sedimentation rate (ESR)) and disease activity, in addition to traditional cardiovascular risk factors, independently affected CVD risk in this patient cohort.^32^ Increased levels of ESR in people with PsA are associated with arterial stiffness, even after adjustment for other cardiovascular risk factors, while elevated CRP and ESR correlate with endothelial dysfunction.^33–35^ Circulating white blood cells and cytokines, for example, interleukin (IL)-17A, have also been noted to be significantly elevated in patients with PsA and CVD.^35,36^ TNF levels are also elevated in people with PsA and CVD, although interestingly, the evidence remains unclear as to whether treatment with TNFis definitively reduces cardiovascular risk in this patient group.^37–39^

The exact mechanisms by which PsA associates so strongly with CVD via this pro-inflammatory state are still being elucidated. However, high-throughput studies of genomics, transcriptomics, proteomics and metabolomics are beginning to shed light on this area.^40^ Metabolomics studies in particular, through metabolite profiling via nuclear magnetic resonance spectroscopy, can identify metabolites across multiple biological pathways, providing a comprehensive view of the overall metabolic state.^41^ This has the potential to improve our understanding of the pathogenesis underlying complex diseases such as CVD and immune-mediated conditions such as PsA, as well as the interplay between them, through isolating markers of inflammation and metabolic syndrome. Such work is, however, in its early stages, and it is likely to be some time before metabolomics can be harnessed for clinical use.

When examining circulating lipoproteins, significantly raised levels of low-density lipoprotein (LDL) and low levels of high-density lipoprotein have been identified in PsA.^42^ The fatty acid, lignoceric acid, which is elevated in patients with PsA compared to psoriasis alone, indicates increased cardiometabolic burden.^43^ PsA is associated with increased levels of oxidative stress and subsequent altered lipid metabolism, perpetuating the development of CVD and related conditions.^44^ Targeted metabolomic profiling by Colaco et al. identified 13 metabolite biomarkers (in addition to age and sex) that demonstrated good performance in predicting cardiovascular risk in a cohort of people with psoriatic disease (639 with PsA, 338 with psoriasis alone), including recognised factors associated with CVD, such as lipoproteins and triglycerides, and others including certain amino acids.^41^ Remnant cholesterol and apolipoprotein B, a primary constituent of LDL, were associated with a 30%–40% increased risk of CVD. Similarly, glycoprotein acetyls, a systemic inflammatory marker associated with circulating leucocytes, TNF and IL-6, is also highly predictive of CVD, in both PsA and psoriasis.^41,45^ Further differences in the metabolome have also been identified in studies comparing people with psoriasis to those with PsA, with levels of inflammatory lipid mediators, especially leukotriene B4 and glycoursodeoxycholic acid, predictive of PsA progression.^46^ Both metabolites are associated with the development of CVD.^47–49^

## Forecasting the future: Assessing and predicting cardiovascular risk in PsA

While investigative techniques such as metabolomics can provide valuable insights into the underlying pathogenesis of PsA and CVD, we are still some way from being able to harness these for the assessment of patients and implementation of personalised medicine. Clinical assessment remains key in identifying our patients with an increased risk of CVD, throughout all stages of PsA.

EULAR has produced recommendations for CVD risk management in patients with inflammatory arthritis, which state that ‘rheumatologists should ensure that CVD risk management is performed in patients with inflammatory joint disease’.^12^ They acknowledge that this may vary between countries and may involve other relevant health professionals and specialties. While the task force does not place the responsibility squarely on the rheumatologist, it does state that the ‘treating rheumatologist should ensure that CVD risk assessment and management is being performed regularly, should record who is performing it and should make sure that the patient is aware of the need for regular risk assessment’. Universally, however, it is important to take a holistic approach to managing CVD risk, through programmes such as smoking cessation, physical activity programmes and monitoring for hypertension and diabetes, to combat the development of the metabolic syndrome and the subsequent development of cardiovascular events.

Population-based risk algorithms have become the mainstay of assessing and identifying cardiovascular risk and ensuring lifestyle and medical interventions and/or monitoring is in place. Such tools include the Framingham Risk Score (FRS) and Cardiovascular Risk Score (QRISK3).^50,51^ The FRS comprises age, gender, hypertension, smoking and cholesterol levels and is used to calculate the risk of developing a major cardiovascular event (myocardial infarction, coronary death or angina) within 10 years, categorised into low-, intermediate- and high-risk groups.^52^ While frequently used, the FRS underestimates the cardiovascular risk at the individual level, especially in the presence of factors such as low socioeconomic status and in women.^53,54^ The underestimation in risk is further exacerbated in the presence of chronic inflammatory conditions such as PsA.^15,55,56^ Cohort studies have demonstrated that the 10-year cumulative incidence of CVD events is almost twice as high as that predicted by the FRS in patients with PsA, with the suggestion that ultrasound assessment for subclinical atherosclerosis may improve risk stratification in this patient population.^15,55^ Similar misclassifications in risk have been identified when using alternate tools including the QRISK score.^56^ Interestingly, a recent study in the Clinical Practice Research Datalink in the UK found CVD risk to be underestimated by FRS and QRISK3 in people with osteoarthritis, as well as those with inflammatory arthritis, suggesting inflammation alone cannot explain the discrepancy.^57^

EULAR suggests the use of the Systematic Coronary Risk Evaluation (SCORE) index, which estimates a 10-year risk of CVD, in countries where no national guidelines are available.^12,58^ Unsurprisingly, this too underestimates cardiovascular risk in people with PsA.^59,60^ Considering the known phenomenon of underestimation of CVD risk in people with inflammatory arthritis when using these established tools, EULAR recommends a multiplication factor of 1.5 when assessing risk in people with rheumatoid arthritis. However, a similar recommendation was not made for other forms of inflammatory arthritis, including PsA, and it remains to be seen whether this will be extended to other conditions. In the meantime, therefore, given the evidence for increased CVD risk in PsA, the use of nationally recommended risk prediction tools, such as those described above, remains the mainstay of assessment for these patients.

## Treatment choice in PsA and CVD: Challenges and complexities

The complex mechanisms underpinning the relationship between PsA and CVD influence the treatment given to people with PsA. One nationwide study in the United States demonstrated nearly one-third of patients with inflammatory arthritis, including PsA, switched or discontinued disease-modifying anti-rheumatic drug (DMARD) therapy following a cardiovascular event.^61^ Several classes of drugs are now available for treating this condition. With this has come increased complexity in prescribing, particularly when considering CVD and related comorbidities such as T2DM and obesity. A systematic review and meta-analysis in 2014, looking at the effect of several drug groups on cardiovascular events in PsA, found inconclusive evidence to suggest non-steroidal anti-inflammatory drugs (NSAIDs) increase the risk of cardiovascular events, although there is a known association with major adverse cardiovascular events (MACE) in the general population.^62,63^ The use of NSAIDs in PsA remains controversial, with one study demonstrating an increased risk of CVD with NSAID use (albeit in the presence of biologic therapy), while another shows a risk reduction.^64,65^

In terms of conventional synthetic DMARDs (csDMARDs), methotrexate is the most commonly prescribed treatment in this category for PsA. Methotrexate reduces systemic inflammation and oxidative stress, conferring beneficial effects for cardiovascular risk in people with PsA.^63,66^ Treatment with methotrexate has also been associated with relatively fast improvement in endothelial function in patients with inflammatory arthritis, including PsA, independent of change in disease activity.^67^

Biologic DMARDs (bDMARDs) are now commonly prescribed to people with moderate to severe PsA and are highly effective in controlling disease activity.^68^ These range from TNFis to IL1-2/23 and IL-17 inhibitors. There is increasing evidence to suggest that TNFi may have net clinical benefits for MACE in PsA, including improvement in endothelial function, carotid intima media thickness and reduced subclinical atherosclerosis.^37,38, 69,70^ Meta-analysis data in a combined cohort of patients with PsA and psoriasis have shown TNFi use to be linked to reducing the incidence of myocardial infarction and an overall decreased mortality rate.^37^ A recent large cohort study, comparing TNFi with apremilast, other bDMARDs, csDMARDs and corticosteroids, showed this group of drugs to be associated with the lowest rates of myocardial infarction, compared to the other groups.^71^ A slight progression in subclinical atherosclerosis has been seen in one small cohort in Italy, following the commencement of TNFi treatment, but the process was noted to decelerate by 5 years.^72^ Overall, therefore, TNFis appear to be beneficial in aiding the risk reduction of CVD in people with PsA.

IL-12/23 and IL-17 inhibitors are now increasingly used in the treatment of PsA and include drugs such as ustekinumab (IL-12/23 inhibitor), secukinumab (IL-17A inhibitor) and ixekizumab (IL-17A inhibitor). Their cardiovascular safety profile remains uncertain. A recent large nationwide study in a French PsA cohort found a small increased risk of MACE (i.e. myocardial infarction or cerebral infarction) with the use of IL-12/23 and IL-17 inhibitors compared with TNFi, but the overall MACE rate remained low.^73^ Reassuringly, an earlier study with similar numbers of patients included showed no difference in the risk of developing atrial fibrillation or MACE when taking ustekinumab compared to TNFi.^74^ A greater number of studies have assessed cardiovascular risk with these medications in people with psoriasis. Interestingly, two meta-analyses on the association of anti-IL-12/23 agents and MACE came to two different conclusions: Ryan et al. found no statistically significant difference in rates of MACE between IL-12/23 inhibitors and TNFi, while Tzellos et al found a significant increased risk, with an odds ratio of 4.23. As well as highlighting potential differences in methodology between the two reviews (which included similar papers), it also reinforces the need for larger and more robust studies to assess the risk of CVD and MACE in this high-risk cohort.^75–77^

In more recent years, targeted synthetic DMARDs, which work via inhibition of the Janus kinase (JAK) pathway, have entered the PsA treatment realm. In the United States, tofacitinib was approved by the Food and Drug Administration (FDA) in 2017 for the treatment of PsA, followed by upadacitinib in 2021.^78–80^ The European Medicines Agency approved tofacitinib and upadacitinib for PsA in 2017 and 2019 respectively. Prior to this, these drugs had been approved for use in rheumatoid arthritis. However, safety concerns gradually emerged following their initial approval and widespread use, especially pertaining to MACE, cancer and serious infections, albeit in studies in RA.^81^ Specific to CVD, It was found that the incidence rate of MACE was higher with tofacitinib compared to TNFi, with a likely elevated risk in older patients and those with a history of smoking, who have a baseline higher CVD risk.^81^ The incidence rate for tofacitinib at a dose of 5 mg twice daily was 0.91 per 100 patient-years, compared to 0.73 per 100 patient-years in patients taking TNFi (HR 1.24; 95% CI 0.81–1.91). Although rheumatoid arthritis and PsA have different pathophysiologies, and patient and cardiovascular risk profiles, this trial understandably had implications for the use of tofacitinib more widely, including in PsA, not least the FDA black-box warning which followed the publication of these results. A recent analysis of adverse event reports obtained from the pharmaceutical company, in which just over 5000 cases of PsA were reviewed, showed comparable rates of all adverse events in patients with PsA and rheumatoid arthritis, including MACE.^82^ Given the findings, caution is advised when considering the use of JAK inhibitors in older people (aged 65 years and over) and those with cardiovascular risk factors or a smoking history.^83^

While not recommended as an optimal long-term treatment option in PsA, corticosteroids are not uncommonly used in this patient cohort for short-term symptomatic relief and during flares.^84,85^ Complications in relation to CVD arising from long-term corticosteroid use are well documented, and in fact cited as a key safety issue in the most recent EULAR guidelines on the management of PsA.^85^ A population-based cohort analysis in the United Kingdom Clinical Practice Research Datalink, comprising 87,794 people with immune-mediated inflammatory diseases, found an increased risk of CVDs associated with glucocorticoid dose intake even at lower doses (<5 mg). This included hazard ratios of 1.69 (95% CI 1.54–1.85) for atrial fibrillation, 1.75 (95% CI 1.56–1.97) for heart failure, 1.76 (95% CI 1.51–2.05) for acute myocardial infarction and 1.78 (95% CI 1.53–2.07) for peripheral arterial disease.^86^ A large systematic review and meta-analysis in patients with rheumatoid arthritis, psoriasis and PsA reported corticosteroid use to be associated with an increased risk of cardiovascular events, including myocardial infarction, stroke and heart failure.^63^ Corticosteroid use is also associated with weight gain, T2DM and hypertension, all associated with CVD. Thus, in addition to their lack of long-term efficacy in PsA, there is ample evidence to avoid the use of corticosteroids where possible in a patient population with an already increased risk of CVD at baseline, especially where other CV risk factors are present.^85,87^

In summary, the choice of drug treatment for PsA is not only influenced by the presence and severity of cardiovascular comorbidities but also may have an impact on the progression of CVD in the patient. This emphasises the need for optimal assessment of cardiovascular risk factors at both baseline and through the disease trajectory, so medication can be adjusted dynamically according to changes in comorbidity status.

## Conclusion

To conclude, our review underscores the critical link between CVD and PsA, highlighting the complex interplay of systemic inflammation, exogenous risk factors and metabolic syndrome, which together contribute to an increased risk of CVD and MACE. A more nuanced understanding of this relationship will pave the way for innovative therapeutic strategies and improved patient-centric care. With the dawn of metabolomics and similar investigative methods, the potential for personalised medicine in the characterisation of individual comorbidity profiles and management, particularly in CVD, seems closer than ever. Advancements in drug therapy and an increasing array of targets provide us with the opportunity to balance cardiovascular risk with treatment benefits when discussing management options with our patients.

We have emphasised the importance of early detection, through risk stratification scores, and intervention strategies, including lifestyle modifications and tailored pharmacological treatments. The management of CVD in people with PsA requires an integrated approach not only involving rheumatologists but also the wider multidisciplinary team, including dermatologists, cardiologists, specialist nurses and pharmacists, to name a few. Such comprehensive evaluation and management of cardiovascular risk factors is likely to lead to better clinical results, reduced risk of MACE and subsequent improvements in patient outcomes and quality of life.

## Declarations

**Ethics approval and consent to participate:** Ethical approval and consent to participate were not required and not applicable to this work. Patient consent was not required for this work, as it comprised a narrative review of existing literature.

**Consent for publication:** Not applicable.

**Availability of data and materials:** Data available upon request.
